# Heart Failure and Preserved Left Ventricular Function: Long Term Clinical Outcome

**DOI:** 10.1371/journal.pone.0041022

**Published:** 2012-07-19

**Authors:** Israel Gotsman, Donna Zwas, Chaim Lotan, Andre Keren

**Affiliations:** Heart Institute, Hadassah University Hospital, Jerusalem, Israel; Universidad Peruana Cayetano Heredia, Peru

## Abstract

**Background:**

Patients with heart failure (HF) have a poor prognosis. The proportion of patients with HF and preserved left ventricular function (LVF) is increasing. Long term prognosis of HF with preserved LVF may not be so benign.

**Objectives:**

To evaluate the long term clinical outcome of patients with HF and preserved LVF and predictors of outcome.

**Methods:**

We prospectively evaluated 309 patients hospitalized with a definite clinical diagnosis of HF. Patients were followed for a mean of 6.5 years for clinical outcome.

**Results:**

More than a third (36%) of the patients had preserved systolic LVF based on echocardiography. The long term survival rate in this group was poor and not significantly different from patients with reduced LVF (28% vs 23% respectively, P = 0.2). The adjusted survival rate by Cox regression analysis was also not significantly different (hazard ratio 1.16, 95% confidence interval 0.87–1.55, P = 0.31). The event free survival from death or heart failure re-hospitalization was also low in both groups and not significantly different between patients with preserved vs. reduced LVF (12% vs. 10% respectively, P = 0.2). Predictors of mortality in patients with preserved LVF were age, functional capacity and serum urea levels.

**Conclusions:**

The long term clinical outcome of patients with heart failure and preserved LVF is poor and not significantly different from patients with reduced LVF.

## Background

Heart failure (HF) is a major epidemic and a significant public health problem [1]. The clinical syndrome of heart failure with preserved left ventricular function (LVF) also defined as HF with a normal ejection fraction, is a common condition in patients with HF and has emerged as a serious clinical problem. A study showed that over the past two decades there has been a significant increase in the number of patients admitted to hospital with heart failure and preserved LVF [2]. The outcome in these patients may be better than in patients with reduced LVF. However, recent studies suggest that the prognosis in these patients is not so benign [2], [3]. We have previously reported the short term outcome of patients with preserved LVF, and shown that they have a poor prognosis [4]. The aim of the present study was to evaluate the long term clinical outcome of patients with heart failure and preserved LVF and compare the outcome to reduced LVF. We also evaluated predictors of outcome in these patients.

## Methods

### Patients

We prospectively enrolled consecutive patients with HF admitted to an Internal Medicine department with a definite diagnosis of HF. HF was not necessarily the primary cause for hospital admission. Diagnosis of HF during hospitalization was made by the treating physician, and was corroborated either by clinical symptoms and signs consistent with HF, reduced left ventricular function (LVF) by echocardiogram or both. Clinical diagnosis of HF was based on typical symptoms and signs consistent with heart failure: orthopnea, paroxysmal nocturnal dyspnea, elevated jugular venous pressure, ankle edema, hepatomegaly or an enlarged cardiac silhouette on chest X-ray. Echocardiograms included were only those that were performed up to 6 months before enrollment. Classification of LVF by echocardiogram was qualitative using a visual assessment of normal, preserved, mild, moderate or severely reduced LVF. We recruited 362 consecutive patients hospitalized with a definite diagnosis of heart failure during a two year period from January 2001 to December 2002. Echocardiographic data was available in 309 (85%) of the patients. There were no significant differences in the clinical parameters between patients with or without echocardiography. Patients with echocardiography were the cohort of this study. The patients were divided into two groups according to the systolic function by echocardiogram: patients with normal or preserved LVF and patients with mild to severely reduced LVF. The study protocol was approved by the Hadassah-Hebrew University Medical Center Institutional Committee for Human Studies. A waiver was obtained from the institutional committee for written informed consent as the study was exclusively observational.

### Hospital Evaluation

Sociodemographic status including place of residence, ethnic background, education, background (concurrent) diseases as documented by the medical records, the causes of admission and drug prescription on discharge were recorded systematically from the medical records during hospitalization. Echocardiography data including measurements of dimensions and intraventricular septal wall thickness were performed according to standard recommendations of the American Society of Echocardiography and were evaluated and verified by qualified personnel. Left ventricular (LV) and right ventricular (RV) systolic function were assessed qualitatively: normal, mild, moderate and severely reduced function. Comprehensive diastolic indices were not available for analysis.

### Follow-up Evaluation

Patients were clinically followed by telephone up to September 2008. Clinical outcome including the number of re-hospitalizations and death were evaluated. Hospitalizations due to HF exacerbation were also recorded. Hospitalizations due to HF exacerbation was based on a primary diagnosis of this condition on the admission hospital records. Mortality was based on data from the National Census Bureau.

### Statistical Analyses

SPSS version 17.0 for Windows (SPSS Inc., Chicago, Illinois, USA) was used in all analyses. The student’s *t* test or Mann-Whitney U test for continuous variables and the Chi-square test for categorical variables was used to examine the bivariate comparisons for each of the demographic or clinical parameters between patients with preserved versus reduced LVF. Clinical predictors were transformed where appropriate. Log_10_ was used for logarithmic transformations. Kaplan-Meier curves, with the log-rank test, were used to compare survival among the two groups. Multivariate Cox regression analysis was used to evaluate independent variables that determined survival. Parameters included in the multivariate Cox regression analysis incorporated all significant clinical and laboratory parameters on univariate analysis as well as drug treatment. Parameters that were included in the multivariate analysis were age, gender, ischemic heart disease, hypertension, diabetes, atrial fibrillation, admission due to HF, serum urea, sodium, hemoglobin, functional capacity and LVF. Proportionality assumptions of the Cox regression models were evaluated by log–log survival curves and with the use of Schoenfeld residuals. Possible interactions were assessed. A p value of <0.05 was considered statistically significant.

## Results

### Clinical Parameters

The study cohort consisted of 309 consecutive patients hospitalized with a definite diagnosis of HF. Follow-up was complete in all patients. Mean follow-up was 6.5 years. 198 had reduced LVF and 111 (36%) had preserved LVF. The demographics, clinical parameters, echocardiographic data and pharmacological treatment on discharge of these patients are presented in Table 1. Patients with preserved LVF were older and were more likely to be females. These patients also had less ischemic heart disease. NYHA Functional class was not significantly different between the two groups. Admission systolic blood pressure and pulse pressure were higher in patients with preserved LVF. Patients with preserved LVF received less beta-blockers, aspirin and digoxin but received more calcium blockers.

**Table 1:** Demographics and clinical characteristics of the patients with CHF.

Demographics and Clinical characteristics	Preserved LVF(N = 111)	Reduced LVF(N = 198)	All(N = 309)	P value
Age (years)	75±12	72±12	73±12	0.07
Male	39 (35%)	126 (64%)	165 (53%)	<0.001
Admission due to HF	66 (59%)	120 (61%)	186 (60%)	0.8
Hospital mean duration (days)	10±8	9±7	9±7	0.3
NYHA FC Class III-IV	79 (71%)	143 (72%)	222 (72%)	0.8
Admission Systolic BP (mmHG)	142±32	132±28	136±29	<0.01
Admission Diastolic BP (mmHG)	75±15	72±13	73±14	0.06
Pulse Pressure (mmHG)	67±23	61±22	63±23	0.02
Admission Pulse (bpm)	81±17	82±21	81±19	0.8
Peripheral Edema	63 (57%)	107 (54%)	170 (55%)	0.6
Pulmonary Congestion - CXR	47 (42%)	93 (47%)	140 (45%)	0.4
Concurrent Illnesses				
Ischemic Heart Disease	61 (55%)	148 (75%)	209 (68%)	<0.001
S/P Myocardial Infarction	20 (18%)	66 (33%)	86 (28%)	<0.01
S/P Coronary bypass surgery	8 (7%)	44 (22%)	52 (17%)	<0.001
Hypertension	68 (61%)	104 (53%)	172 (56%)	0.1
Diabetes Mellitus	42 (38%)	84 (42%)	126 (41%)	0.4
Hyperlipidemia	25 (23%)	61 (31%)	86 (28%)	0.1
Valve Disease	30 (27%)	42 (21%)	72 (23%)	0.2
Atrial Fibrillation	40 (36%)	62 (31%)	102 (33%)	0.4
Peripheral Vascular Disease	6 (5%)	27 (14%)	33 (11%)	0.02
S/P TIA or CVA	10 (9%)	28 (14%)	38 (12%)	0.2
Chronic Obstructive Lung	36 (32%)	49 (25%)	85 (28%)	0.1
**Laboratory Data on Discharge**				
Hemoglobin (g/dl)	11.3±2.1	11.4±1.8	11.3±1.9	0.6
Serum Creatinine (µmol/L)	129 (95–177)	120 (90–162)	134 (96–181)	0.2
Serum Urea (mmol/L)	12.8 (8.9–19.4)	12.1 (8.4–18.2)	13.7 (9.3–19.6)	0.2
Serum Na+ (mEq/L)	139 (135–141)	139 (135–142)	140 (135–141)	0.8
**Echocardiography Data**				
LA size (cm)	5.9±1.0	5.9±0.8	5.9±0.9	0.7
LV EDD (cm)	5.0±0.6	5.9±0.9	5.6±0.9	<0.001
LV ESD (cm)	3.2±0.5	4.8±1.0	4.2±1.1	<0.001
Intraventricular Septum (cm)	1.15±0.21	1.01±0.30	1.06±0.27	<0.01
Severe Mitral Regurgitation	16 (15%)	27 (15%)	43 (15%)	0.7
Reduced RVF	27 (26%)	83 (43%)	110 (37%)	<0.01
Tricuspid inflow PG (mmHg)	42.5±15.4	38.0±13.2	39.8±14.3	0.03
**Pharmacological Treatment on Discharge**				
ACE-inhibitor/ARB	77 (69%)	142 (72%)	219 (71%)	0.6
Beta blockers	30 (27%)	112 (57%)	142 (46%)	<0.001
Spironolactone	32 (29%)	55 (28%)	87 (28%)	0.8
Furosemide	84 (76%)	142 (72%)	225 (73%)	0.9
Digoxin	18 (16%)	52 (26%)	70 (23%)	0.05
Nitrates	41 (37%)	93 (47%)	133 (43%)	0.1
Calcium channel blockers	36 (32%)	34 (17%)	70 (23%)	<0.01
Statins	25 (23%)	61 (31%)	86 (28%)	0.1
Anticoagulants	29 (26%)	41 (21%)	70 (23%)	0.3
Aspirin	47 (42%)	116 (59%)	163 (53%)	0.02
Anti-arrhythmic	24 (22%)	41 (21%)	65 (21%)	0.8

Data is presented as mean ± standard deviation or median (interquartile range) for continuous variables and counts (percentages) for categorical variables. P value by the student T test or Mann-Whitney U test for continuous variables and the Chi-Square Test for categorical variables.

CXR - Chest X ray. LA - left atrium. LV - left ventricle. EDD – end diastolic diameter. ESD – end systolic diameter. RVF – right ventricle function. PG –pressure gradient.

### Echocardiographic Data

Left ventricular end systolic and end diastolic diameters were smaller in patients with preserved LVF (Table 1). The mean intraventricular septum (IVS) was thicker in patients with preserved LVF and a higher proportion of the patients had abnormal IVS thickness (IVS>1.1 cm): 50% vs. 32%, P = 0.02. Right ventricle function was normal in a larger percentage of patients with preserved LVF. The mean pulmonary pressure estimated by the tricuspid inflow pressure gradient was higher in these patients. However, any degree of pulmonary hypertension (>35 mmHg) was common in both groups (80% vs. 72% in preserved vs. reduced LVF respectively, P = 0.1).

### Clinical Outcome

**Figure 1:**
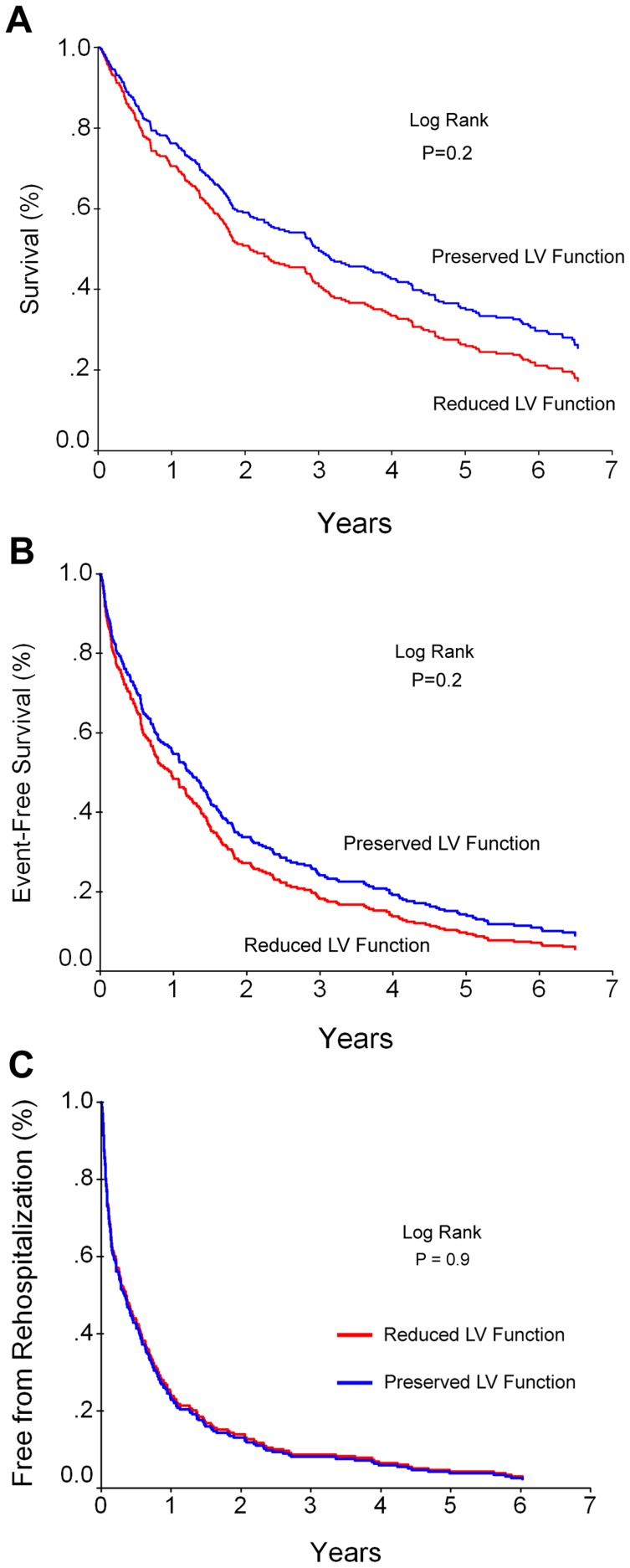
Kaplan Meier survival curves of patients with preserved versus reduced left ventricular function. The survival rate **[A]**, event-free rate of death or heart failure re-hospitalization **[B]** and event-free from all hospitalizations **[C]** of patients with preserved versus reduced LVF (N = 309). There was no significant difference in survival or clinical event-free rate between the groups.

The survival rate in patients with preserved LVF was poor and not significantly different from patients with reduced LVF (28% vs 23% respectively P = 0.2, Figure 1A). The clinical event-free rate that included death or heart failure re-hospitalization was low in both groups and not significantly different between patients with preserved vs. reduced LVF (12% vs. 10% respectively, P = 0.2, Figure 1B). Re-hospitalization event-free rate from any reason was also low and very similar in both groups (13% versus 10% respectively, P = 0.9, Figure 1C).

### Predictors of Outcome

Variables included in the Cox regression analysis were age, gender, IHD, hypertension, diabetes, atrial fibrillation, admission due to HF, serum sodium, urea, hemoglobin, NYHA functional class and LV function. Predictors of increased mortality by multivariate Cox regression analysis in the whole group were age, male gender, functional capacity, hemoglobin level, discharge serum sodium and urea (Table 2). Preserved LVF was not a significant predictor of survival. Exclusion of patients with significant valve disease did not significantly change the results. Inclusion of specific drug therapies in the model did not alter the data. Analysis of predictors in each group separately revealed that significant predictors in patients with preserved LVF were age, functional capacity and serum urea levels (Table 2). Independent predictors of increased death or heart failure re-hospitalization in the whole group were age (hazard ratio (HR) 1.02, 95% confidence interval (CI) 1.01–1.03, P<0.001), hemoglobin level (HR 0.90, 95% CI 0.84–0.96, P<0.01) and NYHA class III/IV (HR 1.90, 95% CI 1.42–2.54, P<0.001). Preserved LVF was not a significant predictor (HR 1.17, 95% CI 0.90–1.52, P = 0.25).

**Table 2:** Predictors of death by Cox Regression analysis.

	Preserved LVF(N = 111)		Reduced LVF(N = 198)		All(N = 309)	
	HR (95% CI)	P value	HR (95% CI)	P value	HR (95% CI)	P value
Age (years)	1.04 (1.02–1.07)	0.001	1.04 (1.02–1.05)	<0.001	1.04 (1.02–1.05)	<0.001
Gender (Male)	1.40 (0.80–2.42)	0.24	1.91 (1.32–2.78)	0.001	1.72 (1.28–2.31)	<0.001
Ischemic Heart Disease	0.54 (0.33–0.89)	0.02	0.99 (0.64–1.53)	0.97	0.78 (0.57–1.06)	0.12
Hypertension	0.82 (0.50–1.37)	0.45	0.89 (0.64–1.23)	0.47	0.86 (0.65–1.12)	0.26
Diabetes Mellitus	0.89 (0.54–1.49)	0.66	1.13 (0.79–1.62)	0.51	1.09 (0.82–1.45)	0.55
Atrial Fibrillation	1.04 (0.64–1.68)	0.88	1.31 (0.91–1.88)	0.15	1.22 (0.92–1.62)	0.17
Admission due to HF	0.90 (0.55–1.47)	0.67	1.00 (0.71–1.42)	0.99	0.97 (0.73–1.28)	0.81
Serum Na<135 mEq/L	1.25 (0.73–2.13)	0.41	1.87 (1.29–2.71)	0.001	1.69 (1.25–2.28)	0.001
Serum Urea* (mmol/L)	1.74 (1.10–2.76)	0.02	1.20 (0.91–1.57)	0.19	1.34 (1.07–1.68)	0.01
Hemoglobin (g/dl)	0.91 (0.79–1.04)	0.16	0.92 (0.84–1.01)	0.08	0.91 (0.84–0.98)	0.02
NYHA FC Class III-IV	2.35 (1.30–4.24)	0.004	1.98 (1.30–3.02)	0.001	1.93 (1.39–2.68)	<0.001
Reduced LVF	/		/		1.16 (0.87–1.55)	0.31

* Log_10_-transformed.

## Discussion

The syndrome of HF has become a major epidemic in the western world, leading to significant morbidity and mortality and it presents a significant global health burden. While systolic dysfunction is considered as the major cause of the syndrome, many of the patients have preserved systolic function. HF with preserved LVF is common and it’s incidence has increased in the last few decades [2]. The present study, based on a hospitalized cohort of patients, showed that 36% of the patients had preserved function. This is similar to most studies demonstrating that 30 to 50% of patients with heart failure have preserved systolic function (LVEF>50%) [2], [5], [6], [7], [8], [9].

The clinical picture of these patients is different from patients with reduced LVF. In our study, the patients were older and were more likely to be females. These patients also had less ischemic heart disease. These characteristics are typical of patients suffering from this syndrome [10]. They also have more hypertension and atrial fibrillation [3], [9] but have less ischemic heart disease [11], [12]. Diastolic dysfunction is commonly found in most of these patients [13] and is probably a major contributory cause of the symptoms.

In the present study, we found that patients with preserved LVF had smaller heart sizes with increased left ventricular hypertrophy as evident by increased IVS thickness. This has been previously reported [7], [14], [15]. Increased wall thickness and LV mass with normal or reduced LV volume is usually present and LV mass/volume ratio was shown to be markedly increased in these patients [14]. Pulmonary pressures in our study were also higher in patients with preserved LVF compared to patients with reduced LVF. Even after exclusion of patients with significant mitral disease or pulmonary disease, mean pressures were increased in these patients. A recent study demonstrated that pulmonary hypertension is common in patients with preserved LVF and predicts mortality [16]. The increased LV wall thickness predisposes these patients to elevated LV end diastolic pressures causing the clinical syndrome of heart failure. It has been established that patients with heart failure and preserved LVF have significant abnormalities in active relaxation and passive stiffness causing decreased LV compliance [17], [18] Decreased LV compliance is associated with a disproportionate elevation of the diastolic pressure, which causes a passive increase in left atrial and pulmonary venous pressures, which produces symptoms of pulmonary venous congestion and increases pulmonary pressures.

Prognosis in patients with preserved LVF was considered to be more benign than in patients with reduced LVF. Reduced EF, particularly below 45% [19] is considered a predictor of a poor outcome in patients with HF. A recent meta-analysis using individual patient data demonstrated that the risk of death did not increase notably until EF fell below 40% [20]. Despite this, the prognosis in patients with HF and preserved LVF is also dismal. Some studies suggest that the survival might be better in such patients; but, the differences are usually not large [2], [9], [11], . Most recent studies demonstrate that although the absolute survival in numbers tends to be slightly better in patients with preserved LVF, this difference is not statistically significant [3], [5], [6], [7], [8], [12]. In the present study we found a very similar result: the survival was slightly better in these patients, however this was not significantly different even after adjustment for background diseases. In addition, significant clinical event rates including death and heart failure rehospitalization was similar with no difference between the groups. We also found that the rate of hospitalization due to any cause was very similar between both groups. The negligible difference in the long term outcome between the two groups was very similar to the short term (1 year) outcome rate in these patients [4]. These findings support the poor prognosis of patients with heart failure and preserved LVF and strengthen the need to find ways to improve prognosis in this common syndrome.

Patients with preserved LVF have a poor prognosis. The precise reasons for this have yet to be determined. This could be due to the syndrome of heart failure or due to comorbid conditions. Data regarding this is limited. A recent study showed that the cause of death was due to heart failure in 46% of these patients, a percentage that was not significantly different from patients with reduced function [24]. Although the clinical characteristics of patients with preserved LVF are quite distinct from patients with reduced LVF, the clinical syndromes are very similar. Furthermore, patients with heart failure and preserved LVF have pathophysiological abnormalities that are qualitatively very similar to patients with reduced LVF. These include hemodynamic changes that increase end diastolic pressures causing reduced exercise performance and activation of the neuroendocrine axis [14]. Reduced functional capacity and increased neurohormonal activation are strong predictors of outcome in patients with HF. It is possible that similar pathophysiological changes predispose patients in both subsets to a similar fate. In the present study, reduced functional capacity was a strong predictor of mortality in patients with preserved as well as reduced LVF.

Treatment modalities for patients with HF and preserved LVF that improve prognosis have yet to be discovered. Traditional therapies with definite benefit in patients with HF and reduced LVF have failed to improve prognosis in patients with preserved LVF. Recently, a small study demonstrated that the aldosterone receptor antagonist spironolactone reduced IVS thickening, inducing favorable cardiac remodeling in diastolic heart failure patients [25]. The beneficial effect of aldosterone receptor antagonism in preserved LVF is presently being tested in randomized clinical trials such as *the Treatment of Preserved Cardiac Function Heart Failure with an Aldosterone Antagonist (TOPCAT) study*.

Limitations of this study: We delineated patients with preserved versus reduced LVF based on qualitative echocardiographical data and not on LV ejection fraction. However, qualitative data of normal or preserved LVF correlates well with an EF above 50% and patients with reduced function have an EF below 50%. Another limitation of this study is that we included patients based on clinical grounds and not on an objective test for HF. Furthermore, only patients hospitalized in internal medicine wards were recruited to this study. Therefore, the results can only be applied to such a patient cohort. In addition, we did not exclude patients with valve disease from this analysis. It is possible that the inclusion of patients with valve disease may be a confounding factor in the analysis; however valve disease was equally present in both groups and exclusion of patients with valve disease demonstrated no significant difference in survival in patients with preserved versus reduced LVF. Also it should be emphasized that there were significant differences in background diseases between the two groups that effect outcome. To overcome this we adjusted for such confounders, however there is a potential for bias despite the adjustments.

In conclusion, the prognosis of patients with clinical heart failure with or without preserved LVF is poor. An improved treatment protocol is needed in patients with HF with preserved LVF.
